# Differential roles of human CD4^+^ and CD8^+^ regulatory T cells in controlling self-reactive immune responses

**DOI:** 10.1038/s41590-024-02062-x

**Published:** 2025-01-13

**Authors:** Xin Chen, Mustafa Ghanizada, Vamsee Mallajosyula, Elsa Sola, Robson Capasso, Karan Raj Kathuria, Mark M. Davis

**Affiliations:** 1 Institute for Immunity, Transplantation, and Infection, Stanford University, Stanford, CA USA; 2 Department of Immunology and Microbiology, University of Copenhagen, Copenhagen, Denmark; 3 Division of Sleep Surgery, Department of Otolaryngology-Head and Neck Surgery, Stanford University School of Medicine, Stanford, CA USA; 4 Department of Microbiology and Immunology, Stanford University School of Medicine, Stanford, CA USA; 5 The Howard Hughes Medical Institute, Stanford University School of Medicine, Stanford, CA USA

**Keywords:** Autoimmunity, Immune tolerance

## Abstract

Here we analyzed the relative contributions of CD4^+^ regulatory T cells expressing Forkhead box protein P3 (FOXP3) and CD8^+^ regulatory T cells expressing killer cell immunoglobulin-like receptors to the control of autoreactive T and B lymphocytes in human tonsil-derived immune organoids. *FOXP3* and *GZMB* respectively encode proteins FOXP3 and granzyme B, which are critical to the suppressive functions of CD4^+^ and CD8^+^ regulatory T cells. Using CRISPR–Cas9 gene editing, we were able to achieve a reduction of ~90–95% in the expression of these genes. *FOXP3* knockout in tonsil T cells led to production of antibodies against a variety of autoantigens and increased the affinity of influenza-specific antibodies. By contrast, *GZMB* knockout resulted in an increase in follicular helper T cells, consistent with the ablation of CD8^+^ regulatory T cells observed in mouse models, and a marked expansion of autoreactive CD8^+^ and CD4^+^ T cells. These findings highlight the distinct yet complementary roles of CD8^+^ and CD4^+^ regulatory T cells in regulating cellular and humoral responses to prevent autoimmunity.

## Abstract (web summary)

Here the authors use a tonsil organoid culture model system to investigate the roles of human CD4^+^ and CD8^+^ regulatory T cells in controlling self-reactive immune responses. CD4^+^ regulatory T cells were stronger regulators of autoreactive B cells, autoantibodies and antigen-specific antibody affinity, whereas CD8^+^ regulatory T cells predominantly controlled expansion of follicular helper cells and autoreactive CD4^+^ and CD8^+^ T cells.

## Main

How the immune system maintains tolerance to self-antigens while having the ability to launch vigorous responses to foreign antigens has been a central question in immunology since Paul Ehrlich first raised it in 1901 (ref. ^1^). After some false starts, the modern era of investigations into this question emerged with analyses of B cell tolerance by Goodnow^2^ and the discovery, by Sakaguchi and colleagues, of a CD4^+^ T cell subset expressing the transcription factor FOXP3 that has been shown to have a major role in preventing autoimmunity^3,4^. More recently, a small subset of CD8^+^ T cells in mice was found by Cantor and colleagues to be another T cell subset involved in controlling autoreactivity^5–7^. Work by our group^8,9^ has shown that these T cells have a critical role in preventing autoimmunity following infection and have elevated levels in human autoimmune diseases. These CD8^+^ T cells express genes encoding members of the Ly49 family in mice and killer cell immunoglobulin-like receptors (KIRs) in humans and are notably active during infectious diseases; evidence suggests that they are specifically tasked with controlling self-reactive T cells that have been activated by infection^8^. Thus, at least two major types of regulatory T (T_reg_) cell maintain tolerance, but their specific roles have not been explored extensively.

To address this question, we used our recently established human tonsil organoid system^10^. In response to stimulation with influenza and other vaccines, this system recapitulates the processes of somatic hypermutation, affinity maturation and B cell maturation and produces antigen-specific antibodies^10,11^. Here, we disabled each of the tonsillar CD4^+^ and CD8^+^ T_reg_ cells by knockout (KO) of key genes related to their functions and gauged their effects on self-reactive B and T cells. Ablating the functions of CD4^+^ T_reg_ cells by knocking out the *FOXP3* gene in tonsillar T cells resulted in production of autoantibodies upon stimulation with a panel of classical autoantigens. This was commensurate with the production of autoantibodies that is a hallmark of *FOXP3* mutations in both humans and mice^12,13^, as well as with severe inflammation^14^. Notably, most tonsil organoids exhibited significant increases in the affinity of antigen-specific antibodies, indicating that FOXP3-expressing T cells also suppress generation of high-affinity antibodies. This could explain why most antibody affinities plateau in about the one-nanomolar range^15^, perhaps owing to cross-reactivity to self-antigens. To attenuate the function of CD8^+^ T_reg_ cells, we knocked out the *GZMB* gene, which encodes granzyme B, a critical effector molecule of these cells^16^. This disruption had only a minor effect on autoantibody production but a much more pronounced effect on self-reactive CD8^+^ and CD4^+^ T cells. This finding for CD4^+^ T cells was correlated with the large increase in follicular helper T (T_FH_) cells seen in mice deficient in CD8^+^ T_reg_ cells^6^, a phenotype we also observed in the tonsil organoids. These results suggest that the increase in T_FH_ cells was due to more self-reactive T cells escaping this tolerance checkpoint. Unexpectedly, we found a notable rise in plasmablasts with *GZMB* KO, but this was dependent on T_FH_ cells and was likely to have been an indirect effect. Last, we discovered a significant sex bias in these T_reg_ cell ablations, with tonsils from women typically giving the strongest autoreactive responses. This bias was also observed in unmanipulated tonsillar B cells. This was consistent with the well-known sex biases seen in many autoimmune diseases^17^ and could be a useful diagnostic to identify women at risk.

We conclude that CD4^+^ and CD8^+^ T_reg_ cells have overlapping yet distinct roles in regulating cellular and humoral responses and preventing autoimmunity. These genetic modifications of the tonsil organoids also demonstrate that we can model key features of how autoreactive B and T cells are controlled and, more broadly, quickly test hypotheses and define mechanisms in a purely human system.

## Results

### Tonsillar and blood T_reg_ cells differ in frequency and phenotype

**Fig. 1:**
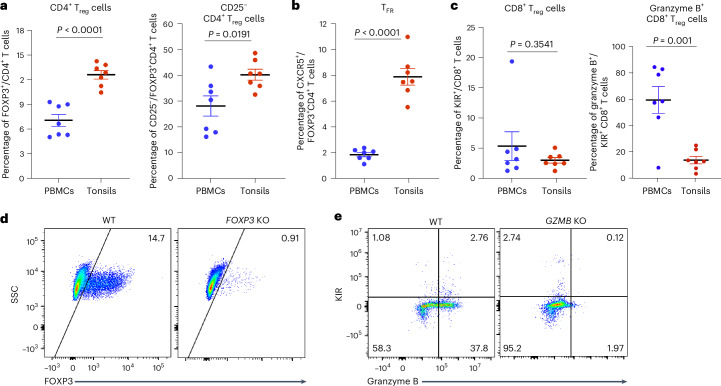
CD4^+^ and CD8^+^ T_reg_ cell subsets in human tonsils showed percentage and phenotypic differences compared with peripheral blood. **a**–**c**, Percentages of CD4^+^ T_reg_ cells (CD4^+^FOXP3^+^) and CD25^−^CD4^+^ T_reg_ cells (**a**), follicular T_reg_ cells (T_FR_; CXCR5^+^FOXP3^+^) (**b**), and CD8^+^ T_reg_ cells (KIR^+^CD8^+^) and granzyme^+^CD8^+^ T_reg_ cells (**c**) from blood and tonsil samples from age- and gender-matched donors (*n* = 7). **d**,**e**, Representative FACS plots of FOXP3 expression in CD4^+^ T cells (**d**) and KIR and granzyme B expression in CD8^+^ T cells (**e**) from WT and KO tonsil cultures 5 days postelectroporation. PBMCs, peripheral blood mononuclear cells; SSC, side scatter. The mean ± s.e.m. is indicated. Significance in **a**–**c** was calculated using a two-sided unpaired *t*-test. Source data

**Extended Data Fig. 1:**
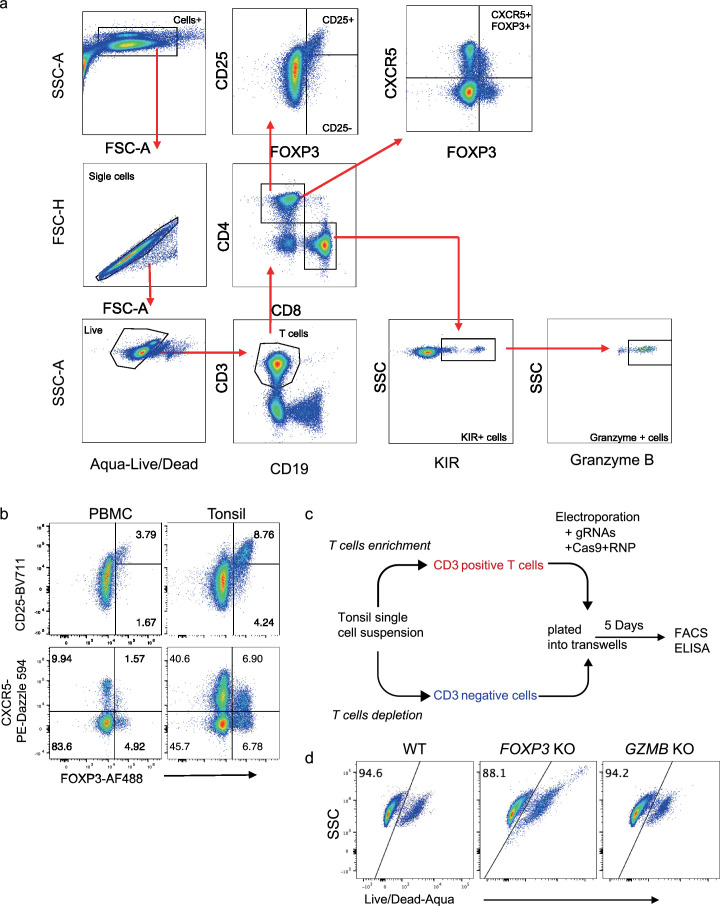
The cell subset percentage and phenotypic differences in tonsils compared to donor-matched peripheral blood and viability after knockouts. **a**, A detailed flow gating strategy is used for the plots in Fig. 1a–c. **b**, A representative FACS profile showing CD25, CXCR5, and FOXP3 expression in PBMC and tonsil samples. **c**, A workflow of generating genetically modified tonsil organoids using Cas9-RNPs. **d**, A representative FACS profile showing Live/Dead staining of T cells from the WT, *FOXP3* KO, and *GZMB* KO tonsil organoids.

CD4^+^ and CD8^+^ T_reg_ cells have been characterized in mice and human blood^14,15^ but less so in human tonsils. To address this gap, we first investigated and compared the percentages and phenotypes of CD4^+^ and CD8^+^ T_reg_ cell subsets in tonsils and peripheral blood cells derived from the same individuals with sleep apnea, with an average age of approximately 40 years. The frequency of FOXP3^+^CD4^+^ T_reg_ cells was significantly higher in tonsils compared with blood (Fig. 1a and Extended Data Fig. 1a). Although circulating CD4^+^ T_reg_ cells usually express high surface levels of CD25 (ref. ^18^), we found that nearly half of the tonsillar CD4^+^ T_reg_ cell population expressed low levels of CD25 (ref. ^19^) (Fig. 1a). In addition, there was a higher percentage of CD4^+^CXCR5^+^FOXP3^+^ follicular T_reg_ cells in the tonsils compared with the blood (Fig. 1b and Extended Data Fig. 1b). The frequencies of CD8^+^ T_reg_ cells expressing KIR were similar in tonsil and blood samples (Fig. 1c). However, CD8^+^ T_reg_ cells from the blood displayed significantly higher levels of granzyme B expression (Fig. 1c).

### Efficient gene KO using Cas9–RNPs

Next, we aimed to ablate the functional T_reg_ cell subsets to breach self-tolerance in human tonsil organoids^10^. Given that cell depletion using the CD25 surface marker becomes inefficient because more than half of the CD4^+^ T_reg_ cell population expresses low levels of CD25, we decided to disrupt the suppressive function of the cells by knocking out key genes *FOXP3* and *GZMB* using Cas9 nucleoprotein (RNP). The *FOXP3* gene encodes an important transcription factor that stabilizes the suppressive function of CD4^+^ T_reg_ cells^20^. *GZMB* encodes granzyme B, which is essential for the cytotoxic function of CD8^+^ T cells^21^ and has been shown to have high transcript and protein expression levels in activated KIR^+^CD8^+^ T cells^22^. We isolated total T cells from human tonsils and electroporated them with RNP complexes consisting of the Cas9 protein, *FOXP3*-targeting guide RNAs (gRNAs) or *GZMB*-targeting gRNAs (Extended Data Fig. 1c). gRNAs consisting of scrambled sequences were used as a control to generate wild-type (WT) T cells. To avoid the possibility of the attached beads interfering with the function of antigen-presenting cells (for example, B cells and myeloid cells), we purified the unlabeled CD3-negative cells separately from a new vial of tonsil cells. The CD3-negative cells and electroporated CD3-positive cells were enumerated, combined and plated into transwells at a density of 6 × 10^6^ cells per organoid as before^10^. After 5 days, we assessed the viability and the KO efficiency by measuring intracellular protein levels via flow cytometry. T cells from WT and KO cultures maintained good viability (90% live cells) (Extended Data Fig. 1d). In *FOXP3* KO tonsil organoids, the intracellular expression of FOXP3 markedly decreased from 14.7% to 0.91% (Fig. 1d). Similarly, in *GZMB* KO cultures, the intracellular expression of granzyme B in KIR^+^CD8^+^ T cells was significantly reduced, achieving an average KO efficiency of 95% (Fig. 1e). These results demonstrate that highly efficient gene KO can be achieved using Cas9–RNPs in human tonsillar T cells, while maintaining favorable cell viability.

### KO tonsil organoids show inflammatory phenotypes

**Fig. 2:**
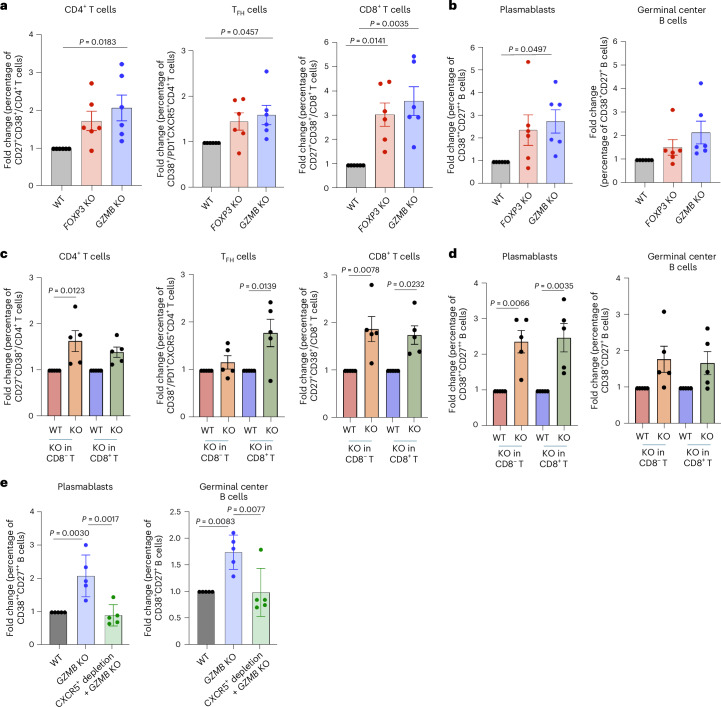
Inflammatory T and B cell phenotypes in *FOXP3* and *GZMB* KO tonsil organoids. **a**,**b**, Statistical analysis of fold change in frequency of activated (CD27^+^CD38^+^) CD4^+^ and CD8^+^ T cells and T_FH_ (CD38^+^PD1^+^CXCR5^+^CD4^+^) cells (**a**) and germinal center B cells and plasmablasts (**b**) in organoids with *FOXP3* KO or *GZMB* KO T cells compared with WT (*n* = 6 donors). **c**,**d**, Statistical analysis of fold change in frequency of activated (CD27^+^CD38^+^) CD4^+^ and CD8^+^ T cells and T_FH_ (CD38^+^PD1^+^CXCR5^+^CD4^+^) cells (**c**) and germinal center B cells (CD38^+^CD27^+^) and plasmablasts (CD38^++^CD27^++^) (**d**) in organoid culture with *GZMB* KO CD8^+^ cells or CD8^−^ T cells compared with WT. **e**, Statistical analysis of fold change in percentage of germinal center B cells and plasmablasts in organoids with WT or *GZMB* KO in CD8^+^ T cells or in those with CXCR5^+^ depletion followed by *GZMB* KO in CD8^+^ T cells compared with WT controls (*n* = 5 donors). Mean ± s.e.m. is indicated. Statistical significance was calculated using one-way analysis of variance (ANOVA) followed by Tukey’s multiple comparisons test. Source data

**Extended Data Fig. 2:**
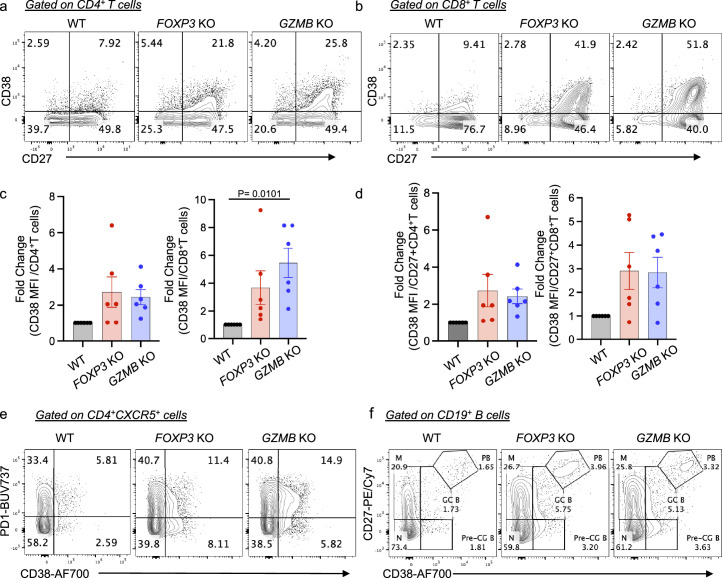
Tonsil organoids with *FOXP3* KO and *GZMB* KO total T cells displayed inflammatory phenotypes after 5-day culture. **a, b**, Representative FACS plots showing the expression of CD38 and CD27 on CD4^+^ T cells (**a**) and CD8^+^ T cells (**b**) in organoids with WT, *FOXP3* KO, and *GZMB* KO T cells. **c, d** Statistical analysis of the fold change in CD38 MFI of CD4^+^ T cells (**c**, left panel) and CD8^+^ T cells (**c**, right panel), as well as CD27^+^CD4^+^ T cells (**d**, left panel) and CD27^+^CD8^+^ T cells (**d**, right panel) in organoids with WT, *FOXP3* KO, and *GZMB* KO T cells (n = 6). **e** Representative FACS plots showing the expression of PD1 and CD38 on Tfh cells (pre-gated on CD4^+^ CXCR5^+^ ) in WT and KO tonsil organoids after 5-day culture without stimulation. **f**, Representative FACS plot showing the expression of CD27 and CD38 on B cells (pre-gated on CD3^-^CD19^+^ ) in WT and KO tonsil organoids after 5-day culture without stimulation. The significance in (**c**) and (**d**) was calculated by one-way ANOVA followed by Tukey’s multiple comparisons test. Source data

To investigate the impact of ablation of the *FOXP3* and *GZMB* genes in T cells on the overall phenotype of tonsil organoids, we examined the percentages of different B and T cell subsets using surface markers associated with activation and differentiation. In the near absence of *FOXP3* or *GZMB* gene in total T cells, tonsil organoids exhibited signs of inflammation, characterized by increases in percentages of cells expressing activation marker CD38 and costimulation molecule CD27 among both CD4^+^ and CD8^+^ T cells compared with WT (Fig. 2a and Extended Data Fig. 2a–d). In addition, there was a significant elevation in the percentage of activated T_FH_ cells from *GZMB* KO tonsil organoids (Fig. 2a and Extended Data Fig. 2e). With both KOs, tonsil organoids showed an increased percentage of germinal center B cells (CD38^+^CD27^+^) and plasmablasts (CD38^++^CD27^++^) (Fig. 2b and Extended Data Fig. 2f) in most donors.

**Extended Data Fig. 3:**
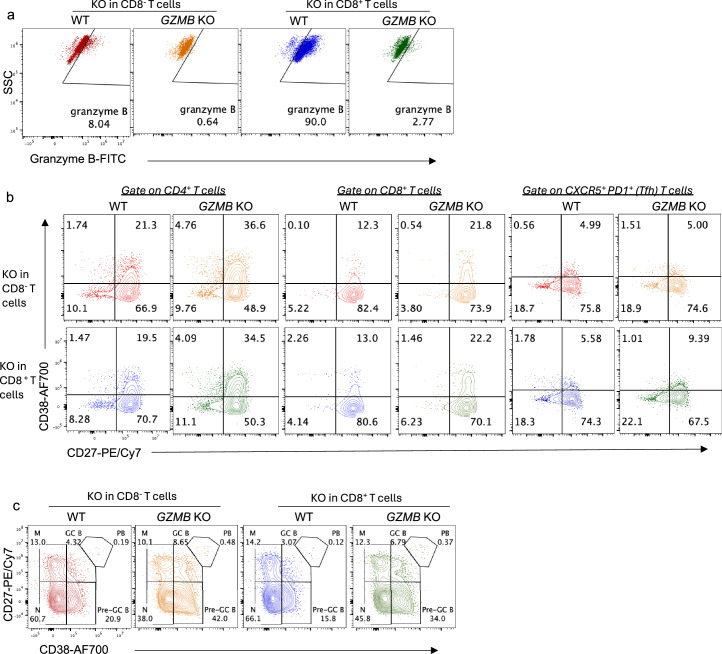
Representative plots showing inflammatory phenotypes in tonsil organoids with GZMB KO in CD8^+^ and CD8^-^ T cells. **a**, Granzyme B expression in CD8^-^ and CD8^+^ T cells 5 days post GZMB KO. **b**, Expression of CD38 and CD27 in CD4^+^ T cells, CD8^+^ T cells and Tfh cells from 5-day cultured tonsil organoids with GZMB KO in CD8^-^ and CD8^+^ T cells. **c**, Expression of CD27 and CD38 in B cells from tonsil organoids with GZMB KO in CD8^-^ and CD8^+^ T cells.

To further investigate the role of CD8^+^ T_reg_ cells in the inflammatory phenotypes observed in organoids with *GZMB* KO T cells—particularly given prior reports of cytotoxic effects mediated by CD4^+^ T_reg_ cells via granzyme B on autoreactive T cells^23^—we replicated experiments by knocking out *GZMB* in either CD8^+^ or CD8^−^ T cells (Extended Data Fig. 3a). Our findings revealed significant expansion of CD4^+^ T cells, T_FH_ cells, CD8^+^ T cells and plasmablasts within tonsil organoids containing *GZMB* KO CD8^+^ T cells (Fig. 2c,d and Extended Data Fig. 3b,c). These results were consistent with the phenotypes seen for total T cells. Notably, *GZMB* KO in CD8^−^ T cells also affected inflammation, as we observed a similar increase in the percentage of the CD27^+^CD38^+^ population, especially in CD4^+^ T cells, CD8^+^ T cells and plasmablasts (Fig. 2c,d and Extended Data Fig. 3b,c).

**Extended Data Fig. 4:**
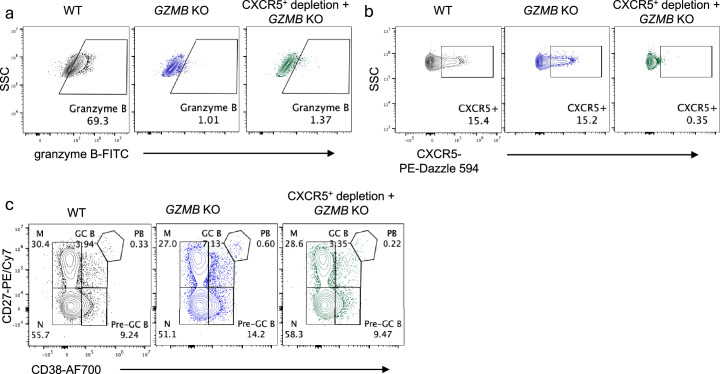
Representative plots showing plasmablast differentiation after CXCR5^+^ depletion and GZMB KO in CD8^+^ T cells of tonsil organoids. **a**, Granzyme B expression in CD8^+^ T cells under the indicated conditions from tonsil organoids. **b**, CXCR5 expression in CD4^+^ T cells under indicated conditions from tonsil organoids. **c**, Expression of CD27 and CD38 in B cells from tonsil organoids with WT, GZMB KO, and CXCR5-depleted GZMB KO CD8^+^ T cells.

T_FH_ cells play essential parts in the formation of germinal centers and direction of B cell differentiation by providing costimulatory signaling^24–26^. To further investigate whether the expansion of plasmablasts we observed in organoids with *GZMB* KO in CD8^+^ T cells was due to direct interaction between T and B cells or occurred indirectly via T_FH_ cells, we examined B cell differentiation after efficient depletion of CXCR5^+^ T cells followed by KO of *GZMB* in CD8^+^ T cells (Extended Data Fig. 4a,b). When CXCR5^+^ T cells were depleted, while the number of CD8^+^ T cells was kept the same as in the WT, there was a significant reduction in the frequency of plasmablasts and germinal center B cells in *GZMB* KO tonsil organoids (Fig. 2e and Extended Data Fig. 4c). This finding suggests that the increased plasmablast levels require the presence of T_FH_ cells and thus are likely to represent an indirect effect.

### Differential autoantibody production in KO tonsil organoids

**Fig. 3:**
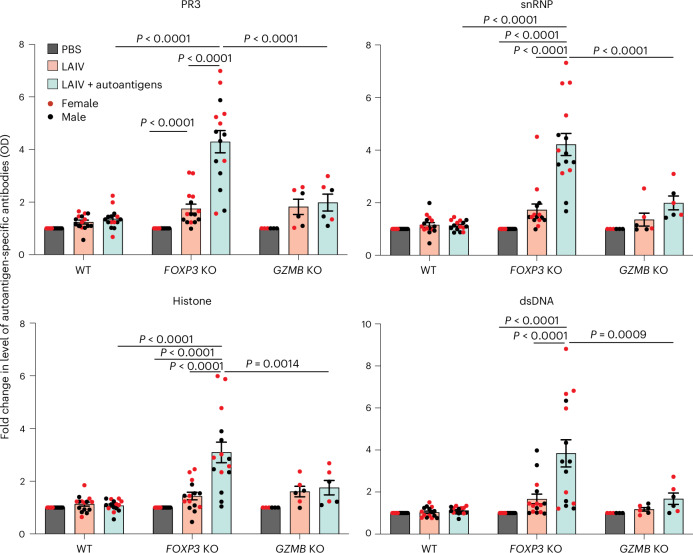
Differential autoantigen-specific antibody production in *FOXP3* KO and *GZMB* KO tonsil organoids after stimulation. Autoantibodies specific to PR3, dsDNA, snRNP and core histone were detected in the supernatant of 10-day cultures under the indicated conditions (*n* = 15 for *FOXP3* KO; *n* = 6 donors for *GZMB* KO). The fold change in optical density (OD) values compared with PBS was calculated. The mean ± s.e.m. is indicated. Statistical significance was calculated by two-way ANOVA followed by Tukey’s multiple comparisons test. Source data

**Extended Data Fig. 5:**
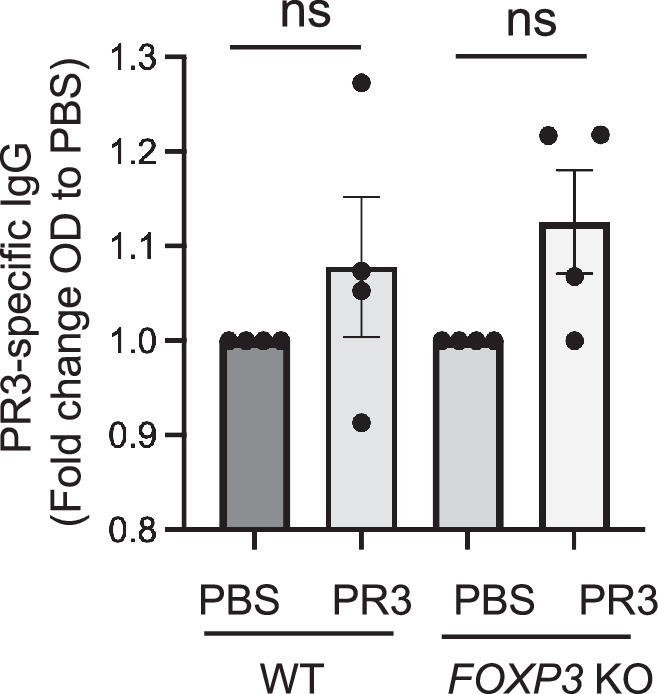
PR3-Specific Autoantibody Production in Organoids with WT or *FOXP3* KO T Cells. Ten-day cell supernatant from organoids with WT or *FOXP3* KO T cells w as collected, and PR3-specific autoantibody was measured by ELISA (n = 4). The fold change in PR3-specific antibody (OD) between PBS and PR3 conditions was calculated. Significance was determined using one-way ANOVA followed by Tukey’s multiple comparisons test. ns, not significant. Source data

**Extended Data Fig. 6:**
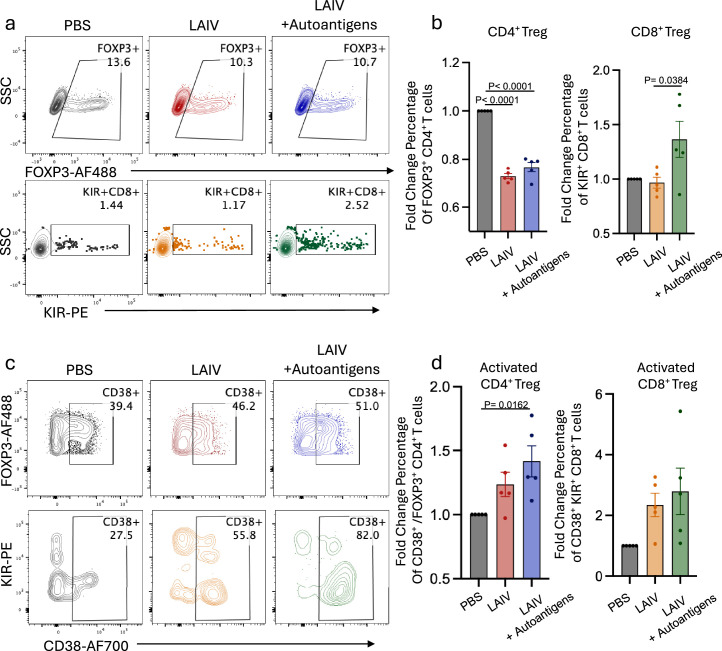
CD4^+^ and CD8 ^+^ Tregs markers and phenotypes in WT organoids stimulated with LAIV or LAIV plus autoantigens. **a**, Representative FACS plots showing CD4^+^ Tregs (*FOXP3*^*+*^ in CD4^+^ T cells) or CD8^+^ Tregs (KIR^+^ in CD8^+^ T cells) in WT tonsil organoids after 5 days of culture. **b**, Fold change in the percentage of FOXP3^+^CD4^+^ T cells and KIR^+^CD8^+^ T cells in WT tonsil organoids stimulated with LAIV or LAIV plus autoantigens (n = 5 donors). **c**, Representative FACS plots showing CD38 expression on CD4^+^ Tregs (pre-gated on FOXP3^+^CD4^+^ T cells) or KIR^+^CD8^+^ Tregs (pre-gated on KIR^+^CD8^+^ T cells) in WT tonsil organoids after 5 days of culture. **d**, Fold change in the percentage of activated CD38^+^ FOXP3^+^CD4^+^ T cells and CD38^+^ KIR^+^CD8^+^ T cells in WT tonsil organoids stimulated with LAIV or LAIV plus autoantigens (n = 5 donors). Significance in (**b**) and (**d**) was calculated by one-way ANOVA followed by Tukey’s multiple comparisons test. Source data

Despite inflammatory phenotypes being observed in B cells within both KO tonsil organoids, there was no significant change in autoantibody production compared with WT tonsil organoids, even with addition of the autoantigen to the culture (Extended Data Fig. 5). Previous studies have suggested that viral infection could be a primary factor in initiation of autoimmune diseases^27^, and mouse models have shown the development of autoimmune phenotypes following viral infection^6^. Thus, we investigated whether live attenuated influenza vaccine (LAIV) could induce an autoimmune response in the germinal center. The WT, *FOXP3* KO and *GZMB* KO tonsil organoids were stimulated with phosphate-buffered saline (PBS), LAIV alone or a combination of LAIV and autoantigen cocktail. The autoantigen cocktail contained proteinase 3 (PR3), core histones, double-stranded DNA (dsDNA) and small nuclear ribonucleoproteins (snRNP), which are relatively common targets of autoantibodies in patients with autoimmune diseases^28–30^ and viral infections^14^. In WT tonsil organoids, LAIV stimulation led to a decreased percentage of CD4^+^ T_reg_ cells and an increased percentage of CD8^+^ T_reg_ cells (Extended Data Fig. 6a,b), both characterized by elevated CD38 expression (Extended Data Fig. 6c,d). However, no significant increase in autoantibodies was observed following LAIV stimulation (Fig. 3). By contrast, the *FOXP3*-deficient tonsil organoids showed up to seven-fold higher levels of autoantibodies compared with WT after stimulation with LAIV and autoantigens (Fig. 3). We also observed that most *FOXP3* KO tonsil organoids with high autoantibody production were derived from female donors, whereas stimulated *GZMB* KO tonsil organoids secreted only minimal levels of autoantibodies. These results suggest that CD4^+^ T_reg_ cells have a more prominent role in control of autoantibody responses compared with CD8^+^ T_reg_ cells, consistent with *FOXP3* deficiencies observed in mice and humans^10,11^.

### Autoreactive B cells in *FOXP3* KO organoids expanded after stimulation

**Fig. 4:**
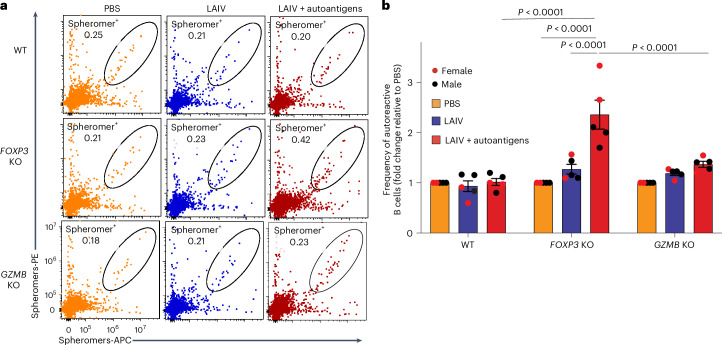
Autoreactive B cells expanded from *FOXP3* KO tonsil organoids after stimulation with LAIV and autoantigens. **a**, Representative FACS plot showing costained spheromer-positive B cells specific to PR3, snRNP-C and core histone (H3.1) after 7 days of culture. **b**, Fold change in the percentage of costained B cells between stimulation and PBS conditions in tonsils with WT, *FOXP3* KO and *GZMB* KO T cells 7 days poststimulation (*n* = 5 donors). The mean ± s.e.m. is indicated. Statistical significance was calculated using two-way ANOVA followed by Tukey’s multiple comparisons test. Source data

The higher levels of autoantibody production in the *FOXP3* KO tonsil organoids led us to hypothesize that the frequency of autoreactive B cells would also be elevated following stimulation. To test this hypothesis, we generated a pool of three ‘spheromers’, which are highly sensitive multimer reagents based on modified maxi-ferritin^31^, specifically targeting the autoantigens PR3, snRNP-C and core histone. In the presence of LAIV, the autoantigen cocktail profoundly stimulated the expansion of self-antigen-specific B cells in *FOXP3* KO tonsil organoids (Fig. 4a,b), consistent with the observed autoantibody levels. Notably, the top two *FOXP3* KO tonsil organoids that produced the higher frequency of autoreactive B cells after LAIV plus autoantigen stimulation were also derived from female donors (Fig. 4b). By contrast, knocking out *GZMB* in tonsillar T cells had only a minor impact on the frequencies of autoreactive B cells (Fig. 4a,b), confirming that CD4^+^ T_reg_ cells modulate autoantibody secretion by constraining the activation of autoreactive B cells.

### Differential autoreactive T cell response in KO organoids

**Fig. 5:**
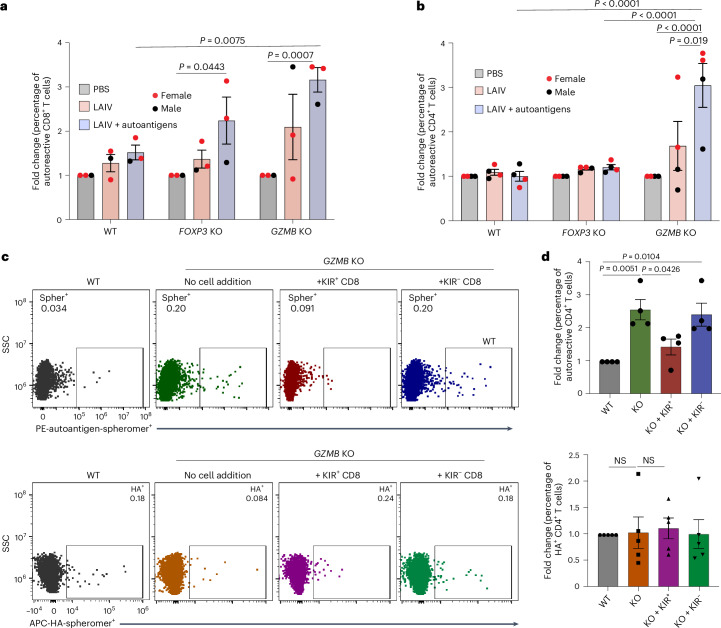
Differential CD4^+^ and CD8^+^ T cell responses to autoantigens in *FOXP3* KO and *GZMB* KO tonsil organoids after stimulation with LAIV and autoantigen stimulation. **a**, HLA-A*0201 tonsil organoids under the indicated WT or KO conditions were cultured for 7 days and then collected for spheromer staining. The percentage of autoreactive CD8^+^ T cells was determined after gating CD8^+^CD3^+^ T cells and human papillomavirus spheromer-negative T cells. The fold change in the percentage of autoreactive T cells under stimulated conditions compared with the control group (treated with PBS) was calculated (*n* = 3 donors). **b**, HLA-DRB1*04:01 tonsil organoids with WT, *FOXP3 KO* or *GZMB* KO total CD3^+^ T cells (*n* = 4 donors) were cultured for 7 days, followed by staining with self-peptide-specific or HA-specific spheromers. The fold change in the percentage of autoreactive-specific CD4^+^ T cells compared with the PBS condition was calculated. **c**, KIR^+^CD8^+^ T cells were depleted, followed by *GZMB* KO in CD8^+^ T cells. Subsequently, KIR^+^CD8^+^ or KIR^−^CD8^+^ T cells were reintroduced into the culture before stimulation with LAIV and autoantigens. Control cultures (WT) received LAIV stimulation without depletion or gene KO. Representative FACS plots show the percentages of spheromer-positive autoreactive CD4^+^ T cells (upper panel) or HA-specific CD4^+^ T cells (lower panel) from tonsil organoids under the indicated conditions. **d**, Fold change in the frequency of autoreactive and HA-specific CD4^+^ T cells compared with WT (*n* = 5 donors). Mean ± s.e.m. is indicated. Statistical significance in **a**, **b** and **d** was calculated using two-way ANOVA followed by Tukey’s multiple comparisons test. Spher, spheromer; NS, not significant. Source data

Although *GZMB* KO tonsil organoids generated a mild autoantibody response (Fig. 3), in most donors we observed an increased percentage of activated T cells compared with WT tonsil organoids (Fig. 2a). This suggests that CD8^+^ T_reg_ cells may exhibit a more robust suppressive response against autoreactive T cell responses. To test this hypothesis, we analyzed the effects of CD8^+^ T_reg_ cells on CD8^+^ T cells specific to self-peptides derived from fructose bisphosphate aldolase, keratin, Y-chromosome-encoded SMCY, preproinsulin (PPI) and glutamic acid decarboxylase 65. These self-peptides have previously been detected in the blood of healthy humans, and SMCY-specific CD8^+^ T cells, in particular, are strictly regulated in males^32^. To detect CD8^+^ T cells specific to these self-peptides, we generated HLA-A*0201 spheromers and also used a peptide from the hepatitis C virus as a negative control. We stimulated the tonsil organoids with WT, *FOXP3* KO and *GZMB* KO T cells derived from HLA-A*0201 donors with PBS, LAIV, or LAIV supplemented with the autoantigen-derived peptides. After 7 days poststimulation, two of three *FOXP3* KO tonsil organoids showed a two-fold increase in the frequency of self-specific CD8^+^ T cells. All three *GZMB* KO tonsil organoids showed an almost-three-fold increase with statistical significance in self-peptide-specific CD8^+^ T cells after stimulation with LAIV and the autoantigen peptide pool (Fig. 5a).

**Extended Data Fig. 7:**
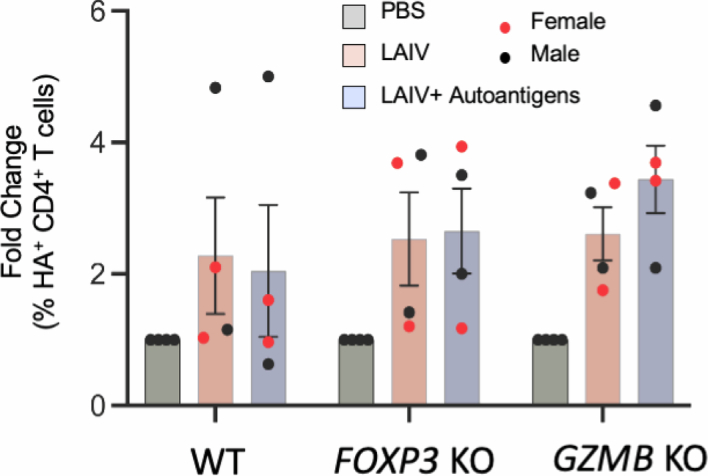
The percentage of HA-specific CD4^+^ T cells in organoids with WT, *FOXP3* KO, or *GZMB* KO T cells. HLA-DRB1*04:01 tonsil organoids with WT, *FOXP3 KO* or *GZMB* KO total CD3^+^ T cells (n = 4 donors) were cultured for 7 days, followed by staining with HA-specific spheromer. The fold change in the percentage of HA-specific CD4^+^ T cells relative to the PBS condition was calculated. Mean ± s.e.m is indicated. Significances were calculated by two-way ANOVA followed by Tukey’s multiple comparisons test. All p-values are greater than 0.05. Source data

We next investigated the regulation of autoreactive CD4^+^ T cells by T_reg_ cell subsets. We selected self-peptides from gp100, fibrinogen or PPI, as these are relevant in antitumor responses and autoimmunity^33–36^. These antigens can be recognized by CD4^+^ T cells in healthy individuals expressing HLA-DRB1*04:01 (ref. ^34^). Influenza hemagglutinin (HA)-specific spheromer reagents were included in the staining panel to assess the suppression by CD4^+^ and CD8^+^ T_reg_ cells of autoreactive T cells compared with other activated T cells. Notably, we observed a statistically significant expansion of CD4^+^ T cells specific to self-antigens, particularly in tonsil organoids with *GZMB* KO T cells stimulated by LAIV and autoreactive peptides (Fig. 5b). Whereas HA-reactive CD4^+^ T cells expanded after LAIV stimulation, there was no significant difference in the percentage of HA-specific CD4^+^ T cells across the WT, *FOXP3* KO and *GZMB* KO cultures (Extended Data Fig. 7). Furthermore, the majority of expanded autoreactive CD4^+^ T cells in the *GZMB* KO stimulated tonsil organoids were derived from female donors (Fig. 5b). By contrast, knocking out *FOXP3* did not affect the frequency of CD4^+^ T cells specific to self-antigens (Fig. 5b).

To further confirm that the increase in autoreactive CD4^+^ T cells from *GZMB* KO organoids was specifically due to functional impairment of KIR^+^CD8^+^ T cells, we depleted KIR^+^CD8^+^ T cells from tonsils derived from DRB1*04:01^+^ donors, followed by KO of *GZMB* in CD8^+^ T cells. KIR^+^ and KIR^−^CD8^+^ T cells were then reintroduced into the culture and supplemented with selected self-peptides from gp100, fibrinogen, or PPI and LAIV cocktail. In the absence of KIR^+^CD8^+^ T cells and granzyme B, the cocktail profoundly stimulated the expansion of autopeptide-specific CD4^+^ T cells. Reintroducing KIR^+^CD8^+^ T cells from the same donor significantly reduced the percentage of autoreactive T cells, whereas introducing an equal amount of KIR^−^CD8^+^ T cells did not (Fig. 5c,d). By contrast, there was no significant effect on the percentage of HA^+^ T cells under comparable conditions. These results suggest that CD8^+^ T_reg_ cells are a potent suppressor of autoreactive CD4^+^ T cells.

Overall, our results demonstrate that CD4^+^ and CD8^+^ T_reg_ cells have distinct areas of responsibility for suppression of self-reactive lymphocytes, albeit with some overlap.

### *FOXP3* KO enhances higher antibody affinity

**Fig. 6:**
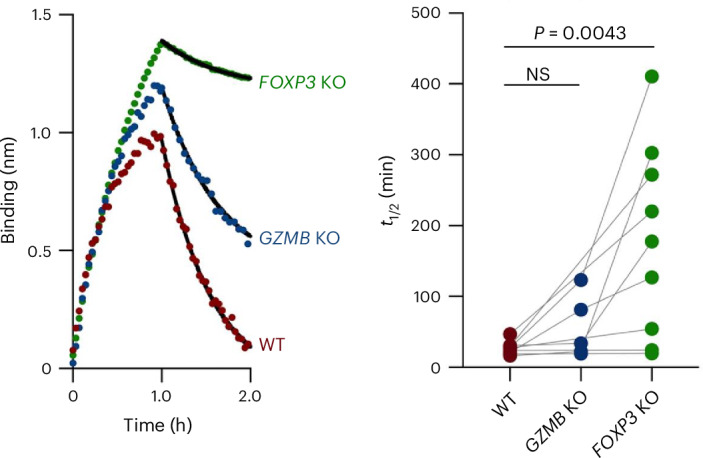
*FOXP3* KO enhances antibody affinity, whereas *GZMB* KO shows only a limited effect. Cell supernatants from LAIV-stimulated WT, *GZMB* KO and *FOXP3* KO tonsil cultures were collected after 10 days of culture. Biolayer interferometry assay was performed to measure the binding affinity of antibodies to HA CA/09 hemagglutinin. An overlay of binding traces under the indicated condition is shown. The *t*_1/2_ was calculated from the off-rates (*k*_off_) using the equation for first-order rate kinetics: *t*_1/2_ = 0.693/*k*_off_. Statistical significance was determined using one-way ANOVA followed by Tukey’s multiple comparisons test. NS, not significant. Source data

As *FOXP3* is critical in regulation of autoantibody production, we investigated whether it could also affect antibody responses against foreign antigens. Taking advantage of the fact that we had used LAIV as an adjuvant in these experiments, we measured the half-life (*t*_1/2_) of the elicited antibodies against recombinant influenza hemagglutinin (HA). The tonsil organoids with WT, *FOXP3* KO or *GZMB* KO T cells were stimulated with PBS or LAIV for 10 days. Supernatants from the organoid cultures were collected for the biolayer interferometry assays. Knocking out *FOXP3* in tonsillar T cells led to an increase in the *t*_1/2_ of the antibodies, such that it was up to 18-fold higher than that in the donor-matched WT condition (Fig. 6), indicating that *FOXP3*-expressing T cells enable production of antibodies with much higher binding affinities in most tonsil donors. The effect of *GZMB* KO was less dramatic, with an average two-fold improvement in five donors. These data indicate that CD4^+^ T_reg_ cells not only regulate autoreactive B cells but also have an impact on B cells in general, effectively limiting their affinity to the typical range, at or near 1 nM.

### Higher autoreactivity in organoids from women compared with men

**Fig. 7:**
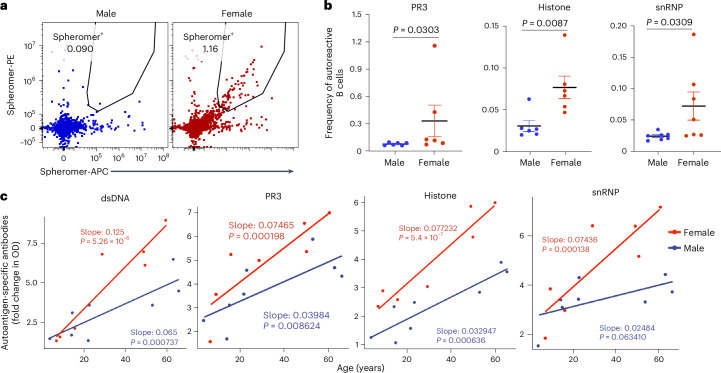
Tonsillar B cells show a higher frequency of autoreactivity in women versus men, regardless of attenuating T_reg_ cells. **a**, Representative FACS plots showing spheromer (PR3)-positive tonsillar B cells from age-matched male and female donors. **b**, Dot plots illustrating the percentage of B cells specific to PR3, histone and snRNP from age-matched donors (*n* = 6). **c**, Autoantibodies specific to PR3, dsDNA, snRNP and core histone were detected by ELISA in the supernatant of 10-day cultures from organoids with WT and *FOXP3* KO T cells (*n* = 15) stimulated with LAIV and autoantigen pools. The fold change in optical density compared with PBS was analyzed using a linear regression model to predict the correlations between autoantibody levels, age and sex. The regression model coefficients (slopes) represent the effect of age on the response variable for each sex, with *P* values indicating statistical significance. Model statistics (*F*-statistic, *P* value and adjusted *R*^2^) are reported for each autoantibody: dsDNA: *F*(2,12) = 30.08, *P* = 2.115 × 10^−5^, adjusted *R*^2^ = 0.806; PR3: *F*(2,12) = 13.97, *P* = 0.0007357, adjusted *R*^2^ = 0.649; histone: *F*(2,12) = 46.49, *P* = 2.231 × 10^−6^, adjusted *R*^2^ = 0.8666; snRNP: *F*(2,12) = 15.58, *P* = 0.0004622, adjusted *R*^2^ = 0.6756. In **b**, significance was calculated using one-sided Mann–Whitney test; mean ± s.e.m. is indicated. Source data

In recent years, risk factors associated with autoimmune diseases, such as sex and aging, have garnered significant attention^37,38^. Using our tonsil organoid system, we explored some factors that might be associated with self-reactive antibodies. A previous study showed that early immature B cells in healthy donors could produce self-reactive antibodies^39^. Thus, we investigated whether autoreactive B cells were also present in tonsils and whether there was a correlation with sex. For this purpose, we used biotinylated spheromers specific to PR3, snRNP or core histone, coupled with PE- or APC-conjugated streptavidin, and costained tonsil samples from age-matched male and female donors. In most donors, we successfully detected B cells specific to these autoantigens, constituting approximately 0.01% to 0.2% of B cells. Notably, these higher levels were found only in the female donors (Fig. 7a,b). Next, we investigated whether sex and chronological age contributed to the autoantibody production we observed in stimulated tonsil organoids with *FOXP3* KO T cells. Indeed, we found a strong correlation between autoantibody production and age, with higher levels of autoantibodies detected in donors above 40 years of age (Fig. 7c). These results highlight the unique and representative nature of tonsil cells and organoids as a valuable system for hypothesis testing and mechanistic studies related to human autoimmune diseases.

## Discussion

Although we have learned a great deal about the characteristics and importance of *FOXP3*-expressing CD4^+^ T cells in enforcing self-tolerance^40–44^, it has only recently emerged that a small subset of CD8^+^ T cells, characterized by Ly49 expression in mice and KIR expression in humans, also plays an important part^7–9^. These CD8^+^ T_reg_ cells are particularly active in the intestines during acute celiac disease and are elevated in patients with other autoimmune diseases, as well as during infections in humans and mice^8^. Conditional KO of the predominant *Ly49* gene in mice leads to autoimmune sequelae following either influenza or lymphocytic choriomeningitis virus infection^8^. The importance of both T_reg_ cell types in controlling self-reactivity raises the question of what their specific roles might be. To address this question, we made use of our recent demonstration that a type of organoid made from human tonsil cells could reproduce many of the hallmarks of an adaptive immune response in vitro, especially when stimulated with various vaccines^10,11^. The single-cell suspensions of tonsil cells as a starting point for organoid culture provide an ideal platform for using CRISPR–Cas9 gene editing to disable CD4^+^ and CD8^+^ T_reg_ cells to analyze their relative roles in controlling autoreactive T and B cells.

Knocking out *FOXP3* resulted in expansion of self-reactive B cells and production of autoantibodies. Autoantibodies are a characteristic of *FOXP3* disruption in humans and mice^45,46^; thus, this effect in the tonsil organoids phenocopies the in vivo results. However, it was surprising that despite both *FOXP3* and *GZMB* KO resulting in similar levels of T_FH_ cells and plasma cells, the latter had only a minimal impact on autoantigen-specific antibody production. Given that CD8^+^KIR^+^ T_reg_ cells are known to target T_FH_ cells, as observed in our study and others^7,47^, but do not affect antibody production, we speculate that the effects of CD4^+^ T_reg_ cells on autoantibody production are exerted through direct interaction with B cells. This is supported by previous work showing that CD4^+^ T_reg_ cells are present in follicles and T–B borders in human tonsils and can directly suppress B cell class switch recombination^48^, as well as inhibiting autoantibody-producing B cells in vitro^49^. Although *FOXP3* ablation in human T_reg_ cells does not affect CD25 and CTLA-4 expression^50^, other signaling molecules may be involved, considering the potential impact of FOXP3 on genome architectural functions and transcriptional regulation^51^. Given the diverse FOXP3^+^ T_reg_ cell population^52^, understanding which subsets contribute to autoantibody production and B cell regulation is crucial.

We also observed substantial increases in the bulk serological affinity of influenza-specific antibodies in most of the *FOXP3*-deficient organoids and to a lesser extent in *GZMB* KOs; this is reminiscent of the increased affinity for self-specific antibodies in *AIRE*-deficient individuals^53,54^ and indicates that disrupting T cell tolerance allows the emergence of higher-affinity antibodies. This is likely to relate to the phenomenon by which the higher the affinity of an antibody, the greater the chances of pathological cross-reactivity to one or more self-antigens^55,56^. This result also suggests a reason that most monoclonal antibodies typically fall within the low nanomolar range and rarely higher^54^.

Our results highlight the differential roles of CD4^+^ and CD8^+^ T_reg_ cells in regulating autoimmunity, potentially by targeting different cellular activities and involvement in different stages of autoimmune development. G*ZMB* KO allowed both autoreactive CD4^+^ and CD8^+^ T cells to proliferate, demonstrating that CD8^+^ T_reg_ cells are the major factor controlling T cell tolerance, especially for autoreactive CD4^+^ T cells. This CD4^+^ effect was earlier observed by the marked increase in T_FH_ cells in mice deficient in CD8^+^ T_reg_ cells^6,8^, suggesting that more T_FH_ cells were allowed to expand than normal when CD8^+^ T_reg_ cell activity was absent or attenuated. In addition, previous work with patients infected with SARS-CoV-2 or influenza has demonstrated increased proportions of CD8^+^ T_reg_ cells during these infections but no change in CD4^+^ T_reg_ cells^8,9^. Infection is a crucial trigger for the breakdown of tolerance, with many autoimmunity patients reporting an infection just before the onset of clinical symptoms^57^. Thus, the disruption of CD8^+^ T_reg_ cell control could be a key first step in triggering clinical autoimmunity. However, as autoantibodies can precede autoimmune disease by years^58–61^, there is likely to be a loss of CD4^+^ T_reg_ cell control earlier.

In summary, we have developed human tonsil organoid models of autoimmunity using CRISPR–Cas9 gene editing techniques. By performing targeted KO, we investigated the distinct roles of two different T_reg_ cell subsets. Knocking out *FOXP3* in the CD4^+^ T_reg_ cell within the tonsil organoids resulted in a drastic increase in autoreactive B cells, autoantibodies and antigen-specific antibody affinity. On the other hand, knocking out *GZMB* in T cells led to dysfunction of CD8^+^ T_reg_ cells, promoting the expansion of T_FH_ cells and autoreactive CD4^+^ and CD8^+^ T cells, and enhancing B cell differentiation. These findings align with observations made in mouse models and patients with defective CD4^+^ and CD8^+^ T_reg_ cells. Further exploration of the tonsil organoid model holds significant potential for identifying new mechanisms and translating them into therapeutic approaches for autoimmune diseases.

## Methods

### Human samples and tonsil processing

The collection and processing of tonsil samples from children and adult volunteers were covered by institutional review board (IRB) protocols 30837 and 60741, approved by the Stanford University IRB. Blood from healthy donors was requested from the Stanford Blood Center under IRB protocol 40146. Written informed consent was obtained from adult participants and from the legal guardians of children aged 0–17 years. No participant compensation was provided for the participants. Any individuals who were taking systemic immunomodulatory drugs, had a history of immunosuppressive or autoimmune diseases, or had a serious active infection at the time of the procedure were excluded.

The procedures for tonsil samples followed those described in a previous study^10^. Tonsil samples were collected immediately after surgery and incubated in Ham’s F-12 medium (Gibco) containing Normocin (InvivoGen), penicillin and streptomycin for at least 1 h at 4 °C before processing. Afterward, tonsil tissue was manually sectioned into small pieces (~5 mm × 5 mm × 5 mm) and passed through a 100-mM filter using a syringe plunger to obtain a cell suspension. The filter was repeatedly washed with complete medium to help filter the cells. Complete medium included RPMI supplemented with Glutamax (Thermo Fisher), 10% fetal bovine serum (FBS), 1× penicillin–streptomycin, 1× Normocin (InvivoGen), 1× insulin–transferrin–selenium supplement (Gibco), 1× nonessential amino acids and 1× sodium pyruvate. Debris was reduced by Ficoll density gradient separation. Collected cells were washed with PBS, enumerated and frozen into cryovials in FBS + 10% dimethyl sulfoxide. Frozen aliquots were stored at −140 °C until use.

### Blood processing

Blood was diluted with cell culture medium and 2% FBS in a 1:1 volume ratio. This diluted blood was then carefully placed on top of a density gradient medium (Ficoll-Paque, GE Healthcare) and centrifuged at 800*g* for 20 min without the brake. For collection of peripheral blood mononuclear cells, a pipette was inserted directly through the upper plasma layer into the interface containing the mononuclear cells. The collected cells were washed twice with medium, frozen in aliquots using FBS with 10% dimethyl sulfoxide and stored at −140 °C until needed.

### Cas9–RNP assembly and electroporation

Cas9–RNPs (Integrated DNA Technologies) were prepared by incubating 20 µM Cas9 with 20 µM gRNA (Synthego) at a 1:1 ratio at 37 °C for 15 min, resulting in a final concentration of 10 µM. The sequences of oligonucleotides are provided in Supplementary Table 1. Electroporation was performed using reagents from a P3 Primary Cell 4D-Nucleofector X Kit S (Lonza) according to the manufacturer’s instructions. Target T cells were gently suspended in P3 buffer supplemented with supplement 1 reagent at a density of 10–20 million cells per 20 μl. The Cas9–RNPs and T cells were mixed gently in the P3 buffer. This mixture was then transferred to a 4D-Nucleofector cuvette from Lonza Bioscience and subjected to electroporation using code EH105. Following electroporation, the cuvette was placed in the tissue culture incubator at 37 °C for 30 min to allow the cells to recover. Once recovered, the cells were ready to be cultured in transwells.

### Cell sorting and organoid assembly

For KO experiments involving total T cells, T cells were isolated from tonsil single-cell suspensions using a Human Pan T Cell Isolation Kit (Miltenyi Biotec) for electroporation. CD3^−^ cells were obtained by depleting CD3^+^ T cells using CD3 MicroBeads, human (Miltenyi Biotec). Organoids were reassembled by combining KO CD3^+^ T cells with CD3^−^ cells at the same donor-specific cell ratio and cultured at a density of 6 × 10^6^ cells in 100 µl per transwell.

For *GZMB* KO experiments in CD8^+^ T cells, untouched CD8^+^ T cells were isolated using a CD8^+^ T Cell Isolation Kit, human (Miltenyi Biotec) for electroporation. The CD8^−^ cell fraction was collected by staining the tonsil single-cell suspension with CD8-PE antibody for 30 min at 4 °C, followed by depletion with anti-PE MicroBeads (Miltenyi Biotec). KO organoids were reassembled by combining CD8^+^ T cells with CD8^−^ cells at the same donor-specific cell ratio and cultured in transwells.

For *GZMB* KO experiments in CD8^−^ T cells, untouched CD3^+^ T cells were first isolated using a Pan T Cell Isolation Kit, human (Miltenyi Biotec), followed by CD8^+^ T cell depletion using a REAlease CD8 MicroBead Kit, human (Miltenyi Biotec). After electroporation and a 30-min recovery, the CD3^−^ cells, CD8^+^ T cells (beads removed according to the manufacturer’s instructions) and *GZMB* KO CD8^−^ T cells were recombined at the same donor-specific cell ratio and cultured at 6 × 10^6^ cells in 100 µl per transwell.

For CXCR5 depletion followed by *GZMB* KO in CD8^+^ T cells, tonsil single-cell suspensions were stained with anti-human CD8-PE (BioLegend) and CXCR5-PE (BioLegend) antibodies at room temperature in the dark for 30 min. PE-positive cells were depleted using anti-PE MicroBeads (Miltenyi Biotec) according to the manufacturer’s instructions. Untouched CD8^+^ T cells were isolated using a CD8^+^ T Cell Isolation Kit, human (Miltenyi Biotec), followed by electroporation. Finally, the KO CD8^+^ T cells were combined with the CD8^−^CXCR5^−^ cell population at the same donor-specific cell ratio.

### Cell culture and stimulation

For culture of cryopreserved cells, aliquots were thawed into complete medium, enumerated and resuspended to 6 × 10^7^ cells per ml for larger cultures or 2 × 10^7^ cells per ml for smaller cultures. Then, 6 × 10^6^ cells in 100 μl were plated into each 12-mm cell culture insert, which had a 0.4-μm pore size (Millipore Sigma). 1 ml complete medium supplemented with 1 ng ml^−1^ IL-21 was added to the lower chamber. 1 μl LAIV (Intranasal FluMist Quadrivalent 2022–2023) was added per well, equivalent to 1.6 × 10^4^ to 1.6 × 10^5^ fluorescent focus units per strain. 1 μg ml^−1^ autoantigens were added in some experiments. 1 μg ml^−1^ of recombinant human B cell-activating factor (BioLegend) may be added to the culture after 4 days. Cell cultures were incubated at 37 °C, 5% CO_2_ with humidity, and supplemented with additional medium to the lower wells as necessary.

### CD8^+^KIR^+^ functional assay

CD8^+^ T cells were purified from tonsil cells using CD8 microbeads (Miltenyi Biotec) per the manufacturer’s instructions and stained with flow antibodies, and live CD8^+^KIR^+^ or CD8^+^KIR^−^ T cells were sorted out by flow cytometry (BD). Sorted KIR^−^CD8^+^ T cells were added back to CD8^−^ tonsil cells, mixed with Cas9 protein and *GZMB* gRNA or scrambled gRNA (as WT) to perform KO. After being allowed to rest for 30 min postelectroporation, equal numbers of KIR^+^ or KIR^−^CD8^+^ T cells were added to the culture of KIR^−^ tonsil cells at a 1:50 ratio. A total of 6 × 10^6^ cells per condition in 100 μl were plated into each 12-mm cell culture insert, which had a 0.4-μm pore size (Millipore Sigma). Then, 1 ml complete medium supplemented with 1 μl LAIV (Intranasal FluMist Quadrivalent 2022–2023, equivalent to 1.6 × 10^4^ to 1.6 × 10^5^ fluorescent focus units per strain) and 10 μg ml^−1^ autopeptides was added to the lower chamber. After day 4, 300 U ml^−1^ of IL-2 and 1 μg ml^−1^ of recombinant human B cell-activating factor (BioLegend) were added to the culture. Cell cultures were incubated at 37 °C, 5% CO_2_ with humidity, and supplemented with additional medium to the lower wells as necessary.

### Flow cytometry

Culture organoids were resuspended by rinsing the membrane with medium and collected from the transwells. Cells were washed with fluorescence-activated cell sorting (FACS) buffer (PBS + 0.1% bovine serum albumin, 0.05% sodium azide and 2 mM EDTA) and treated with Fc receptor block (BioLegend, 10 μg ml^−1^) in FACS buffer for 10 min, followed by staining with live/dead Aqua Zombie stain (Thermo Fisher) and antibodies against surface markers (30 min, 4 °C). Antibodies against surface markers include anti-human CD3 (BUV805, Clone UCHT1, BD) and anti-human CD19 (BUV737, Clone HIB19, BD), anti-human CD4 (BV650, Clone OKT4, BioLegend), anti-human CD8 (BV421, Clone BPA-T8, BioLegend), anti-human CXCR5 (PE-Dazzle 594, Clone J252D4, BioLegend), anti-human PD1 (APC, Clone EH12.2H7, BioLegend), anti-human CD38 (Alexa Fluor 700, Clone HIT2, BioLegend), anti-human CD27 (Pecy7, Clone 0323, BioLegend), anti-human KIR3DL1 (PE, Clone DX9, BioLegend), anti-human KIR2DL2/L3/S2 (PE, Clone DX27, BioLegend), anti-human KIR2DL5 (PE, Clone UP-R1, BioLegend), anti-human CD25 (BV711, Clone M-A251, BioLegend) and anti-human KIR2DL2/L3 (PE, DX27, BioLegend). For intracellular staining, the cells were fixed and permeabilized with the Intracellular Fixation & Permeabilization Buffer Set (eBioscience) followed by staining with anti-human FOXP3 (AF647 or AF488, Clone 259D, BioLegend) or anti-granzyme B antibody (FITC, Clone QA16A02, BioLegend) (30 min, 4 °C). All analyzer data were collected on BD FACSymphony or Agilent NovoCyte Penteon instruments and analyzed using FlowJo (TreeStar) and BD FACSDiva software.

### Antibody detection by enzyme-linked immunosorbent assay

For detection of influenza-specific antibodies, enzyme-linked immunosorbent assay (ELISA) plates (Costar) were coated with 0.1 μg per well of 2022–2023 Fluzone Quadrivalent inactivated influenza vaccine (Sanofi). For detection of autoantigen-specific antibodies, ELISA plates (Costar) were coated with 0.1 μg PR3, snRNP, core histone or dsDNA per well as the capture antigen. Plates were coated with capture antigen overnight, followed by blocking reagents for 2 h. Then, cell supernatants from tonsil culture were added to the coated, blocked plates. After the plates had been washed with washing buffer, horseradish peroxidase-conjugated anti-human secondary antibodies to IgM/IgG/IgA (Sigma) were added to the plate for 1 h, followed by TMB substrate solution (Thermo Scientific). Sulfuric acid was added to stop the reaction, and the plates were read at 450 nm.

### Protein expression, purification and biotinylation

We performed protein production and purification for generating spheromers following a previous study^31^. Briefly, 20 ml of Expi293F cells were subcultured at a density of 3 × 10^6^ viable cells per ml in Expi293 expression medium (Thermo Fisher Scientific) and transfected with expression plasmids using ExpiFectamine 293 transfection reagent according to the manufacturer’s instructions. The cells were supplemented with enhancers after 18 h and further incubated for 4 days. The supernatant was collected by centrifugation (2,000*g*, 30 min, 4 °C) and gently mixed with buffer-equilibrated nickel-nitrilotriacetic acid (Ni-NTA) beads (Qiagen) overnight at 4 °C. Then, the Ni-NTA beads were collected and washed with 20 mM imidazole in HEPES-buffered saline (pH 7.2). The bound protein was eluted using 200 mM imidazole in HEPES-buffered saline (pH 7.2). The proteins were further purified using Amicon Ultra centrifugal units (Millipore Sigma) based on their molecular size. The eluted fractions were analyzed for purity using sodium dodecyl sulfate polyacrylamide gel electrophoresis. The protein was buffer-exchanged to remove the imidazole and biotinylated using a BirA biotin-protein ligase reaction kit (Avidity) according to the manufacturer’s recommendations. The biotinylated proteins were subsequently purified using Amicon Ultra centrifugal units (Millipore Sigma) based on their molecular weight. The eluted fractions were analyzed for purity and biotinylation using sodium dodecyl sulfate polyacrylamide gel electrophoresis.

### Assembly of the peptide-MHC–spheromer complex and autoantigen–spheromer complex

The assembly was started by generating a semisaturated SAv–peptide-MHC_2_ (pMHC_2_) complex. First, 1 μM pMHC-I monomers (NIH tetramer core facility), pMHC-I monomers (NIH tetramer core facility) or autoantigens (generated in-house) were incubated with 0.45 μM streptavidin–fluorophore (BioLegend) at a 1:0.45 ratio at room temperature for 30 min protected from light. The sequences of monomers are provided in Supplementary Table 2. Then, the mixture was incubated with an engineered maxi-ferritin scaffold^31^ at room temperature for 1 h protected from light with gentle rotation. Molar excess of d-biotin was added to saturate any free biotin-binding sites on streptavidin of pMHC_2_. The assembled spheromer was stored at 4 °C for later use.

### Binding affinity measurement

The binding affinity of full-length H1 California/04/2009 influenza hemagglutinin (CA/09 HA) to the antibodies secreted into the culture media of the indicated human tonsil organoids on day 10 after LAIV stimulation was measured by biolayer interferometry using an Octet QK instrument (Pall ForteBio). The antigen (H1 CA/09 HA) diluted in PBST (PBS with 0.05% Tween 20, pH 7.4) was captured using Ni-NTA biosensors. The ligand-bound biosensors were dipped into a serially diluted culture supernatant. The association and dissociation were both monitored for 1 h. Double referencing was performed using unliganded biosensors and an irrelevant *Escherichia coli* maltose-binding protein. The *t*_1/2_ was calculated from the off-rate (*k*_off_) using the equation for first-order rate kinetics, *t*_1/2_2 = 0.693/*k*_off_. Each binding interaction was performed in duplicate.

### Statistical analysis

Most statistical analyses except for those shown in Fig. 7 were performed using GraphPad Prism (v.10). All results are presented as mean ± s.e.m. The significance of the differences between groups were analyzed as described in the figure legends. To assess the relationship between autoantibody levels and donor age and sex, a linear regression analysis was conducted for each autoantibody (dsDNA, PR3, histone and snRNP). Optical density fold changes from ELISA assays were used as the response variable, with age and sex interaction terms as predictors. The model estimated the coefficients (slopes) for age effects in males and females separately. Model fit was evaluated using the *F*-statistic, *P* value and adjusted *R*^2^ value. Statistical significance was determined for each predictor, and residuals were assessed for model accuracy. All analyses were performed using R.

### Reporting summary

Further information on research design is available in the Nature Portfolio Reporting Summary linked to this article.

## Online content

Any methods, additional references, Nature Portfolio reporting summaries, source data, extended data, supplementary information, acknowledgements, peer review information; details of author contributions and competing interests; and statements of data and code availability are available at 10.1038/s41590-024-02062-x.

## Supplementary information

Supplementary Information Donor sex, age and ethnicity reported in the study.

Reporting Summary

Supplementary Table 1 List of genes targeted by gRNA sequences and negative control sequence.

Supplementary Table 2 List of peptide sequences used as epitopes for spheromers to measure percentages of CD8^+^ and CD4^+^ cells from stimulated tonsil organoids.

## Source data

Source Data Fig. 1 Statistical source data.

Source Data Fig. 2 Statistical source data.

Source Data Fig. 3 Statistical source data.

Source Data Fig. 4 Statistical source data.

Source Data Fig. 5 Statistical source data.

Source Data Fig. 6 Statistical source data.

Source Data Fig. 7 Statistical source data.

Source Data Extended Data Fig. 2 Statistical source data.

Source Data Extended Data Fig. 5 Statistical source data.

Source Data Extended Data Fig. 6 Statistical source data.

Source Data Extended Data Fig. 7 Statistical source data.

## Data Availability

All data are available in Figs. 1–7, Supplementary Information and Extended Data Figs. 1–7. Source data are provided with this paper.

## Code availability

Code for statistical analysis using R and existing software packages has been described in the Methods section. No custom code was used in this study.
