# Supplementary material for: Differential roles of human CD4+ and CD8+ regulatory T cells in controlling self-reactive immune responses
Source: Nat Immunol. 2025 Jan 13;26(2):230–9. doi: 10.1038/s41590-024-02062-x (PMC11785521; doi:10.1038/s41590-024-02062-x)
Supplement: Supplementary file 1 — Donor sex, age and ethnicity reported in the study. [file 41590_2024_2062_MOESM1_ESM.pdf]

nature immunology

Supplementary information https://doi.org/10.1038/s41590-024-02062-x

Differential roles of human CD4$^+$ and CD8$^+$ regulatory T cells in controlling self-reactive immune responses

In the format provided by the authors and unedited

Donor ID	gender	Age	Ethnicity/Race
IMD105	F	16	Caucasian
IMD180	M	10	Hispanic/Latino
IMD181	M	4	Unkown
IMD182	M	15	Hispanic/Latino
IMD183	M	17	Hispanic/Latino
IMD191	F	9	Hispanic/Latino
IMD192	F	15	Hispanic/Latino
IMD196	M	12	Hispanic/Latino
PED40	M	14	Unkown
PED44	F	7	Unkown
PED48	M	4	Unkown
PED132	M	8	Unkown
VIP26	F	51	ı-Hispanic/Non-Latino
VIP27	M	35	ı-Hispanic/Non-Latino
VIP33	F	52	Hispanic/Latino
VIP38	F	63	ı-Hispanic/Non-Latino
VIP53	F	63	Asian
VIP57	M	63	ı-Hispanic/Non-Latino
VIP58	M	66	ı-Hispanic/Non-Latino
VIP60	F	59	Hispanic/Latino
VIP63	M	42	ı-Hispanic/Non-Latino
VIP64	F	60	ı-Hispanic/Non-Latino
VIP67	M	40	Hispanic/Latino
VIP68	M	25	Hispanic/Latino
VIP69	M	44	Hispanic/Latino
VIP74	F	19	lon-Hispanic/Latino
VIP76	M	22	Hispanic/Latino
VIP80	F	37	lon-Hispanic/Latino
VIP82	F	49	Hispanic/Latino
VIP84	M	43	lon-Hispanic/Latino
VIP85	F	37	lon-Hispanic/Latino
VIP88	F	29	Unknown
VIP89	F	28	Hispanic/Latino
VIP94	F	38	Hispanic/Latino
VIP95	F	21	lon-Hispanic/Latino
VIP99	F	16	lon-Hispanic/Latino
VIP100	F	50	lon-Hispanic/Latino
VIP102	M	23	Unknown
VIP105	F	39	Asian
VIP106	F	22	Hispanic/Latino

VIP108	M	53	Unknown
VIP124	M	28	Hispanic/Latino
VIP148	M	48	Non-Hispanic/Non-Latino
VIP151	F	32	Non-Hispanic/Non-Latino
VIP156	M	43	Non-Hispanic/Non-Latino
VIP158	M	45	Unknown
VIP159	M	37	Non-Hispanic/non-Latino
VIP173	M	17	Unknown
VIP174	F	48	Unknown
VIP175	M	22	Unknown
VIP179	M	56	Unknown
