# Supplementary material for: Differential roles of human CD4+ and CD8+ regulatory T cells in controlling self-reactive immune responses
Source: Nat Immunol. 2025 Jan 13;26(2):230–9. doi: 10.1038/s41590-024-02062-x (PMC11785521; doi:10.1038/s41590-024-02062-x)
Supplement: Supplementary file 2 — Reporting Summary [file 41590_2024_2062_MOESM2_ESM.pdf]

nature portfolio

Corresponding author(s): Mark M Davis

Last updated by author(s): 2024/12/05

# Reporting Summary

Nature Portfolio wishes to improve the reproducibility of the work that we publish. This form provides structure for consistency and transparency in reporting. For further information on Nature Portfolio policies, see our Editorial Policies and the Editorial Policy Checklist.

## Statistics

For all statistical analyses, confirm that the following items are present in the figure legend, table legend, main text, or Methods section.

| n/a | Confirmed | |
|---|---|---|
| ☐ | ☒ | The exact sample size (*n*) for each experimental group/condition, given as a discrete number and unit of measurement |
| ☐ | ☒ | A statement on whether measurements were taken from distinct samples or whether the same sample was measured repeatedly |
| ☐ | ☒ | The statistical test(s) used AND whether they are one- or two-sided<br>*Only common tests should be described solely by name; describe more complex techniques in the Methods section.* |
| ☒ | ☐ | A description of all covariates tested |
| ☐ | ☒ | A description of any assumptions or corrections, such as tests of normality and adjustment for multiple comparisons |
| ☐ | ☒ | A full description of the statistical parameters including central tendency (e.g. means) or other basic estimates (e.g. regression coefficient) AND variation (e.g. standard deviation) or associated estimates of uncertainty (e.g. confidence intervals) |
| ☐ | ☒ | For null hypothesis testing, the test statistic (e.g. $F$, $t$, $r$) with confidence intervals, effect sizes, degrees of freedom and $P$ value noted<br>*Give P values as exact values whenever suitable.* |
| ☒ | ☐ | For Bayesian analysis, information on the choice of priors and Markov chain Monte Carlo settings |
| ☒ | ☐ | For hierarchical and complex designs, identification of the appropriate level for tests and full reporting of outcomes |
| ☒ | ☐ | Estimates of effect sizes (e.g. Cohen's *d*, Pearson's *r*), indicating how they were calculated |

*Our web collection on statistics for biologists contains articles on many of the points above.*

## Software and code

Policy information about availability of computer code

Data collection: Flow cytometry date were collected using BD FACSDiva software (v7). FACS data were obtained using BD FACSymphony or Agilent NovoVyte Penteon instruments.

Data analysis: FlowJo 10.7.0 was used for the analysis of flow cytometry data. GraphPad Prism 10.1 and R studio 4.0.3 were used for plotting and statistical analysis.

For manuscripts utilizing custom algorithms or software that are central to the research but not yet described in published literature, software must be made available to editors and reviewers. We strongly encourage code deposition in a community repository (e.g. GitHub). See the Nature Portfolio guidelines for submitting code & software for further information.

## Data

Policy information about availability of data

All manuscripts must include a data availability statement. This statement should provide the following information, where applicable:

- Accession codes, unique identifiers, or web links for publicly available datasets
- A description of any restrictions on data availability
- For clinical datasets or third party data, please ensure that the statement adheres to our policy

All data are available in the figures, text, and supplementary figures, with source data.

## Research involving human participants, their data, or biological material

Policy information about studies with human participants or human data. See also policy information about sex, gender (identity/presentation), and sexual orientation and race, ethnicity and racism.

| | |
|---|---|
| Reporting on sex and gender | Both male and female patients are reported. Sex information was obtained from electronic patient records, with informed consent collected from all participants. The patients' ages range from 4 to 66 years, with 64% of the cohort being male and 36% female. Sex data was provided in the source data |
| Reporting on race, ethnicity, or other socially relevant groupings | Race and ethnicity data are listed in the supplementary information |
| Population characteristics | Patients undergoing tonsillectomy were recruited with IRB approval for donating tissues to this study. Patients with serious infections or who were taking systemic immunomodulatory drugs were excluded from the study. The patients' ages range from 4 to 66 years, with 64% of the cohort being male and 36% female. |
| Recruitment | Patients will be referred to the appropriate clinical units for their respective care, such as surgical procedures. Prior to the patient's surgery, our research team will contact the family of the child or adult patient to obtain written informed consent. Patients with serious infections or who were taking systemic immunomodulatory drugs were excluded from the study. Individiuals responsible for consenting patients and collecting tissues were not involved in the scientific aspects of the study. We do not expect any systematic bias in our collection strategy. |
| Ethics oversight | The collection and process of tonsil samples from children and adult volunteers were covered by IRB protocols 30837 and 60741, approved by the Stanford University Institutional Review Board (IRB). Informed consents were obtained from the participants and/or from parents/legal guardians. No participant compensation was provided for the participants. Blood from healthy donors was requested from the Stanford Blood Center under IRB protocol 40146. |

Note that full information on the approval of the study protocol must also be provided in the manuscript.

# Field-specific reporting

Please select the one below that is the best fit for your research. If you are not sure, read the appropriate sections before making your selection.

☒ Life sciences ☐ Behavioural & social sciences ☐ Ecological, evolutionary & environmental sciences

For a reference copy of the document with all sections, see nature.com/documents/nr-reporting-summary-flat.pdf

# Life sciences study design

All studies must disclose on these points even when the disclosure is negative.

| | |
|---|---|
| Sample size | Sample sizes varied from n=4 to n=15 depending on the experiments performed. Sample size was based on previous expeiments (PMID: 33432170) and clinical samples availability. |
| Data exclusions | No data were excluded |
| Replication | The number of donors tested is provided in the figure legends. We anticipated inter-donor variation in response to the stimulation and treatment and responses to stimulation were reported in figures or in text. Different donors were used to demonstrate the range of possible responses. |
| Randomization | Experimental groups were stimulation-related, sex and age-related, thus randomization is not relevant. |
| Blinding | Blinding is irrelevant for this study as it is not a clinical trial. The objective is to assess cell culture responses to various treatments using established assays. |

# Reporting for specific materials, systems and methods

We require information from authors about some types of materials, experimental systems and methods used in many studies. Here, indicate whether each material, system or method listed is relevant to your study. If you are not sure if a list item applies to your research, read the appropriate section before selecting a response.

## Materials & experimental systems

| n/a | Involved in the study |
|---|---|
| ☐ | ☒ Antibodies |
| ☐ | ☒ Eukaryotic cell lines |
| ☒ | ☐ Palaeontology and archaeology |
| ☒ | ☐ Animals and other organisms |
| ☒ | ☐ Clinical data |
| ☒ | ☐ Dual use research of concern |
| ☒ | ☐ Plants |

## Methods

| n/a | Involved in the study |
|---|---|
| ☒ | ☐ ChIP-seq |
| ☐ | ☒ Flow cytometry |
| ☒ | ☐ MRI-based neuroimaging |

# Antibodies

Antibodies used

anti-human CD3 (BUV805) 1/50 BD Biosciences Cat# 612895; RRID:AB_2870183
anti-human CD19 (BUV737) 1/50 BioLegend Cat# 741829; RRID:AB_2871164
anti-human CD4 (BV650) 1/50 BioLegend Cat# 317436; RRID:AB_2563050
anti-human CD8 (BV421) 1/50 BioLegend Cat# 301036; RRID:AB_10960142
anti-human CXCR5 (PE-Dazzle 594) 1/50 BioLegend Cat# 356928; RRID:AB_2563689
anti-human PD1 (APC) 1/20 BioLegend Cat# 329908; RRID:AB_940475
anti-human CD38 (Alexa Fluor 700) 1/100 BioLegend Cat# 303524; RRID:AB_2072781
anti-human CD27 (Pecy7) 1/100 BioLegend Cat# 302838; RRID:AB_2561919
anti-human CD158e1 (KIR3DL1) (PE) 1/50 BioLegend Cat# 312708; RRID:AB_2249498
anti-human CD158b (KIR2DL2/L3, NKAT2) (PE) 1/100 BioLegend Cat# 312606; RRID:AB_2130554
anti-human CD158f (KIR2DL5) Antibody (PE) 1/100 BioLegend Cat# 341304; RRID:AB_2130701
anti-FOXP3 (Alexa Fluor 488) 1/50 BioLegend Cat# 320212; RRID:AB_430887
anti-FOXP3 (Alexa Fluor 647) 1/50 BioLegend Cat# 320214; RRID:AB_492984
anti-granzyme B (FITC) 1/100 BioLegend Cat# 372206; RRID:AB_2687030
anti-human CD25 (BV711) 1/50 BioLegend Cat# 356138; RRID:AB_2632781
anti-human CD8 (PE) 1/10 BioLegend Cat#; 344706; RRID:AB_1953244
anti-human CXCR5 (PE) 1/25 BioLegend Cat# 356904; RRID:AB_2561813

Validation

All antibodies were purchased from commercial suppliers (BD Biosciences, BioLegend, ThermoFisher). Validation statement were confirmed from the manufacturers' website for their relevant used in the study:
anti-human CD3 (BUV805) https://www.bdbiosciences.com/en-us/products/reagents/flow-cytometry-reagents/research-reagents/single-color-antibodies-ruo/buv805-mouse-anti-human-cd3.612895
anti-human CD19 (BUV737) https://www.bdbiosciences.com/en-us/resources/scientific-resources?resourceType::all=all
anti-human CD4 (BV650) https://www.biolegend.com/en-ie/products/brilliant-violet-650-anti-human-cd4-antibody-7786?GroupID=BLG5901
anti-human CD8 (BV421) https://www.biolegend.com/en-ie/products/brilliant-violet-421-anti-human-cd8a-antibody-7152
anti-human CXCR5 (PE-Dazzle 594) https://www.biolegend.com/en-ie/products/pe-dazzle-594-anti-human-cd185-cxcr5-antibody-9860
anti-human PD1 (APC) https://www.biolegend.com/en-ie/products/apc-anti-human-cd279-pd-1-antibody-4413
anti-human CD38 (Alexa Fluor 700) https://www.biolegend.com/en-ie/products/alexa-fluor-700-anti-human-cd38-antibody-6050
anti-human CD27 (Pecy7) https://www.biolegend.com/en-us/products/pe-cyanine7-anti-human-cd27-antibody-8434
anti-human CD158e1 (KIR3DL1) (PE) https://www.biolegend.com/en-us/products/pe-anti-human-cd158e1-kir3dl1-nkb1-antibody-2286
anti-human CD158b (KIR2DL2/L3, NKAT2) (PE) https://www.biolegend.com/en-us/products/pe-anti-human-cd158b-j-kir2dl2-l3-s2-antibody-2281
anti-human CD158f (KIR2DL5) Antibody (PE) https://www.biolegend.com/en-us/products/pe-anti-human-cd158f-kir2dl5-antibody-5809
anti-FOXP3 (Alexa Fluor 488) https://www.biolegend.com/en-us/products/alexa-fluor-488-anti-human-foxp3-antibody-2908
anti-FOXP3 (Alexa Fluor 647) https://www.biolegend.com/en-us/products/alexa-fluor-647-anti-human-foxp3-antibody-2909
anti-granzyme B (FITC) https://www.biolegend.com/en-us/products/fitc-anti-human-mouse-granzyme-b-recombinant-antibody-14430
anti-human CD25 (BV711) https://www.biolegend.com/en-us/products/brilliant-violet-711-anti-human-cd25-antibody-13762
anti-human CD8 (PE) https://www.biolegend.com/en-us/products/pe-anti-human-cd8-antibody-6247
anti-human CXCR5 (PE) https://www.biolegend.com/en-us/products/pe-anti-human-cd185-cxcr5-antibody-8358

# Eukaryotic cell lines

Policy information about cell lines and Sex and Gender in Research

| | |
|---|---|
| Cell line source(s) | Expi293F Cells from thermofisher (cat# A14527) |
| Authentication | Identity of the cell lines were frequently checked by their morphological features |
| Mycoplasma contamination | Mycoplasma contamination was tested negative for mycoplasma via qPCR performed by ThermoFisher |

| Commonly misidentified lines (See ICLAC register) | No commonly misidentified cell lines are used in this study |
|---|---|

## Plants

| | |
|---|---|
| Seed stocks | N/A |
| Novel plant genotypes | N/A |
| Authentication | N/A |

## Flow Cytometry

### Plots

Confirm that:

☒ The axis labels state the marker and fluorochrome used (e.g. CD4-FITC).

☒ The axis scales are clearly visible. Include numbers along axes only for bottom left plot of group (a 'group' is an analysis of identical markers).

☒ All plots are contour plots with outliers or pseudocolor plots.

☒ A numerical value for number of cells or percentage (with statistics) is provided.

### Methodology

| | |
|---|---|
| Sample preparation | Culture organoids were resuspended by rinsing the membrane with media and collected from the transwells. Cells were washed with FACS buffer (PBS+0.1% BSA, 0.05% sodium azide and 2mM EDTA) and treated with Fc receptor block (Biolegend, 10ug/ml) in FACS buffer for 10 mins followed by staining with live/dead Aqua Zombie stain (1/100), and antibodies against surface markers (30min, 4°C). For intracellular staining, the cells were fixed and permeabilized followed by staining with anti-FOXP3 or anti-granzyme B antibody (30min, 4°C). |
| Instrument | FACS data were obtained using BD FACSymphony or Agilent NovoVyte Penteon instruments. BD FACSAria was used for Cell sorting |
| Software | Flow cytometry date were collected using BD FACSDiva software (v7). |
| Cell population abundance | The purify of the sorted cells was more than 95% |
| Gating strategy | For generating the plots in Fig. 1, we began by excluding debris through gating on FSC-A vs. SSC-A. Next, we gated for single cells using a diagonal plot of FSC-A versus FSC-H to remove doublets, followed by selecting live cells by gating the negative population from the Live/Dead stain. We then identified CD3+ T cells, distinguishing them from non-T cells by excluding CD19 + B cells. At this stage, we separated the two main T cell subsets, CD4+ and CD8+ T cells, into distinct populations using a CD4 vs. CD8 plot.<br>To identify the percentage of CD4+ Tregs as shown in Fig 1(A, left panel), we gated on total FOXP3+ cells within the CD4+ population. The percentage of the CD25- population in Fig 1(A, right panel) was further determined by gating on the CD25 negative population from FOXP3+CD4+ T cells. To determine the percentage of T Follicular Regulatory cells, we examined the percentage of CXCR5 and FOXP3 double-positive cells from CD4+ T cells.<br>Within the CD8+ T cell population, we identified CD8+ Tregs based on the expression of KIR (Killer Immunoglobulin-like Receptor), which defines a regulatory subset within CD8+ T cells. Finally, we assessed the Granzyme B expression in these KIR + CD8+ Tregs. |

☒ Tick this box to confirm that a figure exemplifying the gating strategy is provided in the Supplementary Information.
